# Accelerated Evolution of the *ASPM* Gene Controlling Brain Size Begins Prior to Human Brain Expansion

**DOI:** 10.1371/journal.pbio.0020126

**Published:** 2004-03-23

**Authors:** Natalay Kouprina, Adam Pavlicek, Ganeshwaran H Mochida, Gregory Solomon, William Gersch, Young-Ho Yoon, Randall Collura, Maryellen Ruvolo, J. Carl Barrett, C. Geoffrey Woods, Christopher A Walsh, Jerzy Jurka, Vladimir Larionov

**Affiliations:** **1**Laboratory of Biosystems and Cancer, National Cancer InstituteBethesda, MarylandUnited States of America; **2**Genetic Information Research Institute, Mountain ViewCaliforniaUnited States of America; **3**Department of Neurology, Howard Hughes Medical Institute and Beth Israel Deaconess Medical CenterBoston, MassachusettsUnited States of America; **4**Laboratory of Molecular Carcinogenesis, National Institute of Environmental Health SciencesResearch Triangle Park, North CarolinaUnited States of America; **5**Harvard University, CambridgeMassachusettsUnited States of America; **6**St. James's University HospitalLeedsUnited Kingdom

## Abstract

Primary microcephaly (MCPH) is a neurodevelopmental disorder characterized by global reduction in cerebral cortical volume. The microcephalic brain has a volume comparable to that of early hominids, raising the possibility that some MCPH genes may have been evolutionary targets in the expansion of the cerebral cortex in mammals and especially primates. Mutations in *ASPM*, which encodes the human homologue of a fly protein essential for spindle function, are the most common known cause of MCPH. Here we have isolated large genomic clones containing the complete *ASPM* gene, including promoter regions and introns, from chimpanzee, gorilla, orangutan, and rhesus macaque by transformation-associated recombination cloning in yeast. We have sequenced these clones and show that whereas much of the sequence of *ASPM* is substantially conserved among primates, specific segments are subject to high Ka/Ks ratios (nonsynonymous/synonymous DNA changes) consistent with strong positive selection for evolutionary change. The *ASPM* gene sequence shows accelerated evolution in the African hominoid clade, and this precedes hominid brain expansion by several million years. Gorilla and human lineages show particularly accelerated evolution in the IQ domain of *ASPM*. Moreover, *ASPM* regions under positive selection in primates are also the most highly diverged regions between primates and nonprimate mammals. We report the first direct application of TAR cloning technology to the study of human evolution. Our data suggest that evolutionary selection of specific segments of the *ASPM* sequence strongly relates to differences in cerebral cortical size.

## Abstract 2

Mutation of the *ASPM* gene is associated with abnormally small brain size. Comparison of the *ASPM* gene from several primate species suggests it as a target of evolutionary selection for increased brain size

## Introduction

The human brain, particularly the cerebral cortex, has undergone a dramatic increase in its volume during the course of primate evolution, but the underlying molecular mechanisms that caused this expansion are not known. One approach shedding light on the molecular mechanisms of brain evolution is the analysis of the gene mutations that lead to defects in brain development. Among the best examples of such defects is the human primary microcephaly syndrome. Primary microcephaly (MCPH) is an autosomal recessive neurodevelopmental disorder in which the brain fails to achieve normal growth. The affected individuals have severe reduction in brain size; however, the gyral pattern is relatively well preserved, with no major abnormality in cortical architecture (McCreary et al. 1996; Mochida and Walsh 2001). Moreover, there are no recognizable abnormalities in the organs other than the central nervous system. The most common cause of MCPH appears to be mutations in the *ASPM* gene (Roberts et al. 2002).

The *ASPM* gene encodes a 10,434-bp-long coding sequence (CDS) with 28 exons, and spans 65 kb of genomic DNA at 1q31. *ASPM* contains four distinguishable regions: a putative N-terminal microtubule-binding domain, a calponin-homology domain, an IQ repeat domain containing multiple IQ repeats (calmodulin-binding motifs), and a C-terminal region (Bond et al. 2002). Though the exact function of the human *ASPM* in the brain needs to be clarified, the homologue in the fruit fly, *Drosophila melanogaster,* abnormal spindle *(asp),* is localized in the mitotic centrosome and is known to be essential for both the organization of the microtubules at the spindle poles and the formation of the central mitotic spindle during mitosis and meiosis. Mutations in *asp* cause dividing neuroblasts to arrest in metaphase, resulting in reduced central nervous system development (Ripoll et al. 1985; do Carmo Avides et al. 2001; Riparbelli et al. 2001). In the mouse *(Mus musculus)* brain, the *Aspm* gene is expressed specifically in the sites of active neurogenesis. Expression in the embryonic brain was found to be greatest in the ventricular zone, which is the site of cerebral cortical neurogenesis (Bond et al. 2002). This expression profile suggests a potential role for *Aspm* in regulating neurogenesis.

Interspecies comparisons of *ASPM* orthologs have shown their overall conservation, but also a consistent correlation of greater protein size with larger brain size (Bond et al. 2002). The increase in protein size across species is due mainly to the increased number of IQ repeats, suggesting that specific changes in *ASPM* may be critical for evolution of the central nervous system.

In an attempt to reconstruct the evolutionary history of the *ASPM* gene, we isolated large genomic clones containing the entire *ASPM* gene in several nonhuman primate species. Sequence analysis of these clones revealed a high conservation in both coding and noncoding regions, and showed that evolution of the *ASPM* gene might have been under positive selection in hominoids. These clones could also provide important reagents for the future study of *ASPM* gene regulation in its native sequence context.

## Results

### Comparison of Genomic Organization of the *ASPM* Genes in Primates

**Figure 1:**
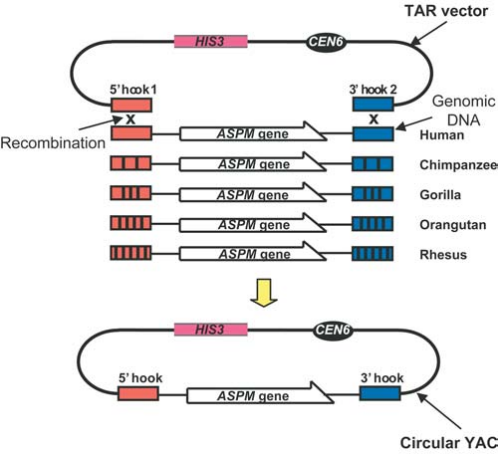
Isolation of the Syntenic Genomic Regions Containing the *ASPM* Gene from Human, Chimpanzee, Gorilla, Orangutan, and Rhesus Macaque by TAR Cloning The method exploits a high level of recombination between homologous DNA sequences during transformation in the yeast Saccharomyces cerevisiae. For isolation, genomic DNA is transformed into yeast spheroplasts along with a TAR vector that contains targeting hooks homologous to the genomic DNA sequence. *CEN* corresponds to the yeast Chromosome VI centromere; *HIS3* is a yeast selectable marker. Recombination between the vector and the genomic DNA fragment results in cloning of the gene/region of interest as YAC. Chromosomal regions with sizes up to 250 kb can be isolated by TAR cloning. For cloning purposes, TAR vector was designed containing a 5′ hook specific to exon 1 and a 3′ hook specific to the 3′ end of the human *ASPM*. Transformation experiments were carried out with freshly prepared spheroplasts for each species. To identify *ASPM*-containing clones, the transformants were combined into pools and examined by PCR for the presence of the unique *ASPM* sequences not present in the vector. The yield of *ASPM*-positive clones from primate species was the same as that from the human DNA (3%). Because the TAR procedure produces multiple gene isolates, six independent TAR isolates for each species were checked. The detectable size of the cloned material corresponded to that predicted if the entire *ASPM* gene had been cloned, i.e., all gene-positive clones contained circular YACs with approximately 65-kb DNA inserts. *Alu* profiles for each species were determined and found to be identical for each species, suggesting that the isolated YACs contained nonrearranged genomic segments. Finally the YACs were retrofitted into BACs, and their restriction patterns were examined by three restriction endonuclease digestions. No differences between *ASPM* clones for each species were found.

Homologues from chimpanzee *(Pan troglodytes),* gorilla *(Gorilla gorilla),* orangutan *(Pongo pygmaeus),* and rhesus macaque *(Macaca mulatta)* were isolated by transformation-associated recombination (TAR) cloning in yeast *(Saccharomyces cerevisiae),* the technique allowing direct isolation of a desirable chromosomal region or gene from a complex genome without constructing its genomic library (Kouprina and Larionov 2003). The method exploits a high level of recombination between homologous DNA sequences during transformation in the yeast. Since up to 15% divergence in DNA sequences does not prevent selective gene isolation by in vivo recombination in yeast (Noskov et al. 2003), for cloning purposes, a TAR vector was designed containing short human *ASPM*-gene-specific targeting hooks specific to the exon 1 and 3′ noncoding regions (see “Materials and Methods”). The TAR cloning scheme for isolating the *ASPM* gene homologues from nonhuman primates is shown in Figure 1. The yield of *ASPM*-positive clones from chimpanzee, gorilla, orangutan, and rhesus macaque was the same as that from the human DNA, suggesting that most homologous regions from nonhuman primates can be efficiently cloned by in vivo recombination in yeast using targeting hooks developed from human sequences.

**Figure 2:**
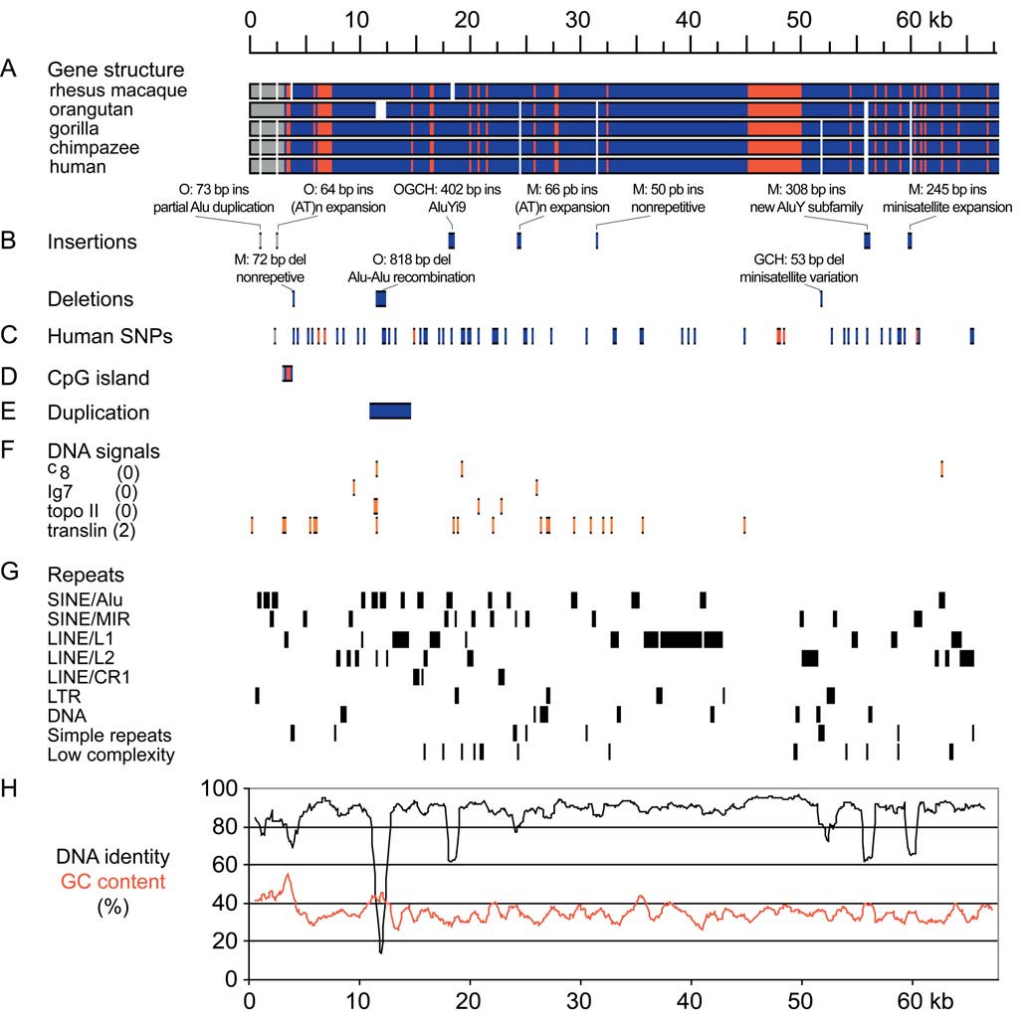
Structure and Evolution of the *ASPM* Gene in Primates The scale of all plots corresponds to the consensus sequence obtained based on a multiple alignment of five *ASPM* genes. (A) Schematic representation of the alignment. Promoter regions, exons, and introns are marked in gray, red, and blue, respectively. White segments correspond to gaps. (B) Positions of long (50 bp or longer) insertions/deletions. “O” denotes orangutan, “M” macaque, “OGCH” the orangutan–gorilla–chimpanzee–human clade, and “GCH” the gorilla–chimpanzee–human clade. (C) Positions of polymorphic bases derived from the GenBank single nucleotide polymorphism (SNP) database. (D) Positions of the CpG island. The approximately 800-bp-long CpG island includes promoter, 5′ UTR, first exon, and a small portion of the first intron. (E) Location of an approximately 3-kb-long segmental duplication. (F) Positions of selected motifs associated with genomic rearrangements in the human sequence. Numbers in parentheses reflect number of allowed differences from the consensus motif (zero for short or two ambiguous motifs, two for longer sites). (G) Distribution of repetitive elements. The individual *ASPM* genes share the same repeats except of indels marked in (B). (H) DNA identity and GC content. Both plots were made using a 1-kb-long sliding window with 100-bp overlaps. The GC profile corresponds to the consensus sequence; the individual sequences have nearly identical profiles.

We have compared complete gene sequences from primate species with a 65-kb, full-size human *ASPM* gene. All the analyzed genes are organized into 28 exons encoding a 3,470–3,479-amino-acid-long protein. *ASPM* genes start with an approximately 800-bp-long CpG island, that harbors promoter sequences, 5′ untranslated regions, and the first exon (Figure 2). *ASPM* sequences share a high degree of conservation (Figure 2H), and pairwise DNA identity ranges from 94.5% for macaque and gorilla to 99.3% for the human–chimpanzee comparison (Table 1). Multiple alignment of the genes revealed a low proportion of indels. Only ten insertions/deletions equal to or longer than 50 bp have been found, all of them located within introns (Figure 2B). Seven detected insertions were mainly associated with repetitive DNA: two *(AT)_n_* microsatellite expansions, three *Alu* insertions, including retroposition of *AluYi9* in the orangutan–gorilla–chimpanzee–human clade, and retroposition of a new macaque-specific *AluY* subfamily similar to human *AluYd2*. Analysis of eight different macaque individuals showed that this particular insertion is polymorphic in the macaque population (data not shown), and thus the insertion appears to be very recent. One macaque-specific 245-bp-long insertion is linked to expansion of a 49-bp-long, minisatellitelike array. The remaining macaque-specific insertion (50 bp) is nonrepetitive. A closer analysis suggests that the insert is not a processed pseudogene of known genes (data not shown).

**Table 1:**
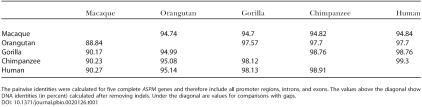
Pairwise Identity of Aligned Primate *ASPM* Genes

The pairwise identities were calculated for five complete *ASPM* genes and therefore include all promoter regions, introns, and exons. The values above the diagonal show DNA identities (in percent) calculated after removing indels. Under the diagonal are values for comparisons with gaps

Of the two detected deletions, the macaque-specific 72-bp-long deletion appears to be associated with nonrepetitve DNA. The second one, an 818-bp-long deletion in orangutan, was probably caused by homologous *Alu–Alu* recombination (see below and Figure S1). The remaining indels are related to expansion/contraction of a short minisatellite array. It was caused either by a 53-bp expansion in the gorilla–chimpanzee–human clade or by two independent deletions/contractions in the macaque and orangutan lineages.

An approximately 3-kb-long intronic segment between exons 4 and 5 is present in several copies in the genome (Figure 2E; Figure S2). Closer analysis of the human genome confirmed that copies of this region are homologous to 24 segmental duplications located mainly in telomeric regions of Chromosomes 1–8, 10, 11, 16, 19, 20, and Y. Based on the sequence similarity and the presence of an *L1P4 LINE* insertion at the 5′ end, the most closely related are three duplications at 7q11–13. The most similar copy is located on Chromosome 7 and shares 93% identity with the *ASPM* intronic segment. Five duplications are located on Chromosome 1; the closest copy is found 27 Mb away from the *ASPM* gene.

We looked for several common motifs associated with genomic breakpoints in cancers (Abeysinghe et al. 2003). Figure 2F shows the positions of such potentially unstable oligonucleotides. Interestingly, the orangutan-specific deletion (Figure 2B) has its 5′ breakpoint located just 1 bp upstream of a sequence 100% identical to the chi-like consensus motif GCWGGWGG (see Figure S1). The chi motif is recognized by the RecBCD-mediated recombination pathway in prokaryotes and seems to be associated with rearrangements in the human genome (Dewyse and Bradley 1991; Chuzhanova et al. 2003). Both deletion breakpoints in the orangutan deletion are located within 5′ parts of two *Alu* sequences, suggesting that the deletion was created by homologous *Alu–Alu* recombination. Similar homologous recombinations with breakpoints located near chi-like motifs in 5′ regions of *Alu* sequences were described previously (Chen et al. 1989; Rudiger et al. 1995).

In summary, despite the presence of a few indels, coding and noncoding regions of *ASPM* homologues show a marked degree of conservation.

### Evolution of the ASPM Protein

**Figure 3:**
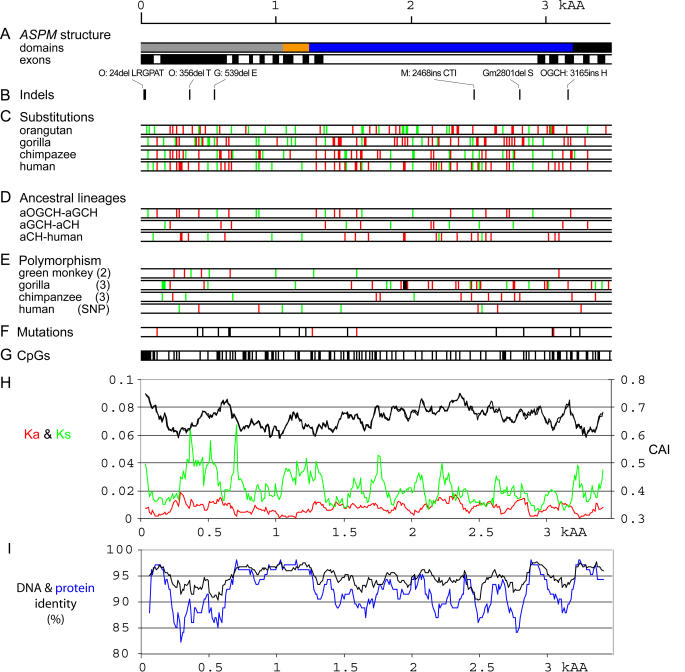
Structure of *ASPM* CDSs and Evolution in Primates The scale of all plots corresponds to the 3,480-amino-acid-long protein alignment; positions in the CDS were scaled accordingly. (A) Structure of the human *ASPM* CDS and protein. The first scheme shows positions of major domains in the ASPM protein (Bond et al. 2002). The putative microtubule-binding domain is in gray, the calponin-homology domain in orange, IQ repeats in blue, and the terminal domain in black. Positions of exons in the CDS are drawn in the second block. To separate individual exons, odd numbered exons are colored in black and even numbered ones in white. (B) Positions of insertions/deletions in the protein sequences. Coordinates correspond to the human protein sequence. “O” denotes orangutan, “G” gorilla, “M” macaque, “Gm” African green monkey, and “OGCH” the orangutan–gorilla–chimpanzee–human clade. (C) Substitutions in hominoid CDSs relative to the common ancestor. The expected ancestor CDS was derived using ML codon reconstruction implemented in PAML. African green monkey and rhesus macaque were outgroups. Nonsynonymous/synonymous (ω = Ka/Ks) ratios were free to vary in all branches. Positions marked in green correspond to synonymous changes relative to the ancestral sequence; the red bars indicate nonsynonymous changes. (D) Synonymous (red) and nonsynonymous (green) changes in ancestral lineages leading to human. aOGCH–aGCH is the ancestral lineage from the orangutan divergence to the gorilla divergence; aGCH–aCH represents the lineage from the gorilla divergence to the chimpanzee common ancestor. aCH–human corresponds to the human lineage after the chimpanzee divergence. There are seven synonymous and 19 nonsynonymous human-specific substitutions. Methods and description are the same as in (C). (E) Positions of polymorphic bases for different CDSs of African green monkey, gorilla, chimpanzee, and human. Positions marked in green correspond to synonymous polymorphisms, and the red bars indicate nonsynonymous sites. Numbers of compared sequences are in parentheses; in the case of human we show nine polymorphic positions (four synonymous and five nonsynomous) from the GenBank SNP database. *ASPM* mutations detected in MCPH patients are shown separately in (F). (F) Positions of 19 mutations reported for MCPH patients (Bond et al. 2002; Bond et al. 2003). All the reported mutations introduce premature stop codons. Mutation sites located within CpG dinucleotides are highlighted in red. (G) Positions of CpG dinucleotides in the human CDS. (H) Comparison of Ka and Ks rates with codon adaptation index (CAI). Ka and Ks values are for all branches (fixed ω ratio); CAI is an average for all five primates (note that CAI differences are very small between the five species). The window was set to 300 bp (100 amino acids) with a 30-bp (10-amino-acid) step. (I) Conservation at the nucleotide and protein level in primates. Y-axis corresponds to proportions of conserved (identical) positions in the CDS and the protein alignment. The plot was obtained using 100-amino-acid-long, overlapping windows, and the step was set to 10 amino acids. In the case of CDS conservation, the window was 300 bp and step 30 bp.

We have analyzed *ASPM* CDSs from six primate species: human, chimpanzee, gorilla, orangutan, rhesus macaque, and African green monkey *(Cercopithecus aethiops)*. Except for orangutan and rhesus macaque, two or more *ASPM* CDSs were used for analysis. ASPM proteins showed the same overall length and domain structure (Figure 3A). The IQ repeat domain contains the same number of repeats, suggesting that their expansion occurred in early primate evolution. The CDSs are, as expected, more conserved than the complete gene sequences with promoter and intronic regions (Table 2; Table 3). Only six short indels were identified (Figure 3B).

**Table 2:**
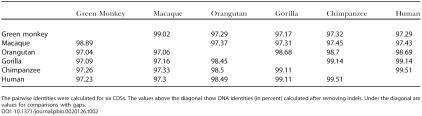
Pairwise Identity of *ASPM* CDSs

The pairwise identities were calculated for six CDSs. The values above the diagonal show DNA identities (in percent) calculated after removing indels. Under the diagonal are values for comparisons with gaps

**Table 3:**
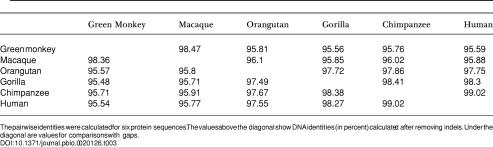
Pairwise Identity of ASPM Proteins

The pairwise identities were calculated for six protein sequences. The values above the diagonal show DNA identities (in percent) calculated after removing indels. Under the diagonal are values for comparisons with gaps

**Figure 4:**
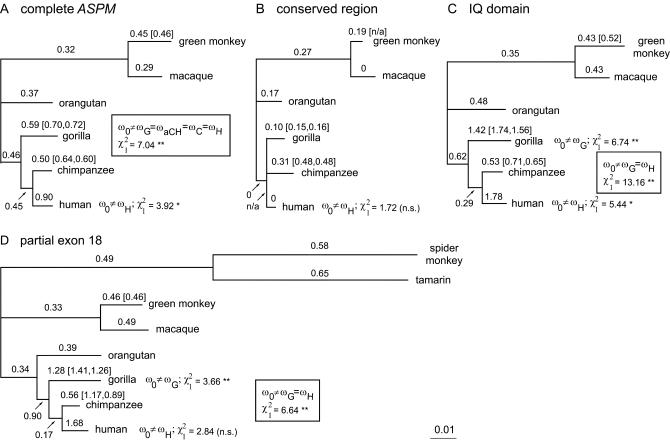
Phylogenetic Trees and ω ratio for Complete *ASPM* and Three Selected Segments Trees and ω (Ka/Ka) ratios were computed using the ML method for codons implemented in PAML. Branch lengths represent ML distances for codons, i.e., using both synonymous and nonsynonymous nucleotide sites, and in all branches the ω ratio was set free to vary. All trees are drawn to the same scale. Branch labels mark the ω ratios for corresponding branches. Values in square brackets show ω for additional cDNA sequences whenever available. Default values and branch lengths were calculated from genomic copies. Selected tested hypotheses are listed. ω_H_ stands for the ω rate in the human lineage, ω_C_ in the chimpanzee lineage, ω_CH_ in the common human–chimpanzee ancestral lineage after the gorilla divergence, ω_G_ in the gorilla lineage, and ω_0_ in all other branches. Single asterisks indicate *p* < 0.05, χ^2^_1_ = 3.84; double asterisks indicate *p* < 0.01, χ^2^_1_ = 6.63. (A) Phylogeny for the complete *ASPM* CDS. In addition to testing different ω values in the human lineage, we also tested the hypothesis that the complete gorilla–chimpanzee–human clade evolved at a constant rate, different from the rest of the tree (compared to the one-ratio model, boxed). (B) The *ASPM* phylogeny derived from a conserved segment from exon 5 to the beginning of the IQ domain (amino acids 676–1,266). The branch connecting the human and chimpanzee common ancestor with the gorilla–chimpanzee–human common ancestor had no substitutions, therefore the ω ratio could not be calculated. (C) IQ domain (amino acids 1,267–3,225). We also tested the hypothesis that the gorilla and human lineages evolved at the same ω rate, different from the rest of the tree (compared to the one-ratio model, boxed). (D) Phylogeny of eight primate sequences from a 1,215-amino-acid-long segment of exon 18 (amino acids 1,640–2,855). We also tested the hypothesis that the gorilla and human lineages evolved at the same ω rate, different from the rest of the tree (compared to the one-ratio model, boxed).

From the DNA and protein conservation profiles (Figure 3I), *ASPM* segments evolve differently along the length of the CDS. N- and C-terminal regions and the region corresponding to exons 5–15 are conserved. In contrast, exons 3 and 4 and the complete IQ repeat domain (positions 1,267–3,225) are more variable. Indeed, nonsynonymous substitutions in hominoid primates (Figure 3C) and in ancestral lineages (Figure 3D) and nonsynonymous polymorphism (Figure 3E) are nearly absent in the conserved central (exons 5–15) and C-terminal regions. This pattern indicates different rates of evolution along the ASPM protein, visualized by plots of synonymous Ks and nonsynonymous Ka rates (Figure 3H) and supported by phylogenetic analysis (see below and Figure 4). It is notable that the comparison of the primate and mouse proteins also revealed the same pattern of conservative and nonconservative regions along ASPM protein (Figure S3).

Analysis of the nonsynonymous/synonymous substitution ratio (ω = Ka/Ks) revealed an elevated value in the human branch (Figure 4A). According to the likelihood ratio test, the human ω rate is significantly different from the rate in the rest of the tree (*p* < 0.05). Also the model that the complete gorilla–chimpanzee–human clade is evolving at one ω rate different from that in the rest of the tree is well supported (*p* < 0.01). Because *ASPM* consists of regions with different degrees of sequence conservation (see Figure 3), we separately analyzed a conserved region (exons 5–15 plus a small part of exon 16) and a variable IQ repeat domain. As can be seen (Figure 4B) the conserved region has all branches shorter, indicating overall a slower rate of evolution. In the human lineage, the ω ratio equals zero; however, the test for whether the human branch has a different (lower) ω rate than the rest did not yield significant values. In contrast, the tree based on the variable IQ repeat domain exhibits ω values greater than one for the human and gorilla branches (Figure 4C). The likelihood ratio test supports the model in which human and gorilla lineages evolved under a significantly higher ω ratio than the rest of the tree. Similar results were obtained for exon 18 with additional sequences from two New World monkeys (Figure 4D). As seen from Figure 4A–4D, different sequences from African green monkey, gorilla, and chimpanzee individuals result in different ω values for their corresponding terminal branches. One chimpanzee sequence also produced an ω ratio greater than one for exon 18 (Figure 4D). It is worth noting that neither codon bias nor selection on third codon positions seemed to influence the synonymous rate Ks strongly (Table S1). Therefore, the high Ka/Ks ratios in human and gorilla are likely to be products of adaptive evolution.

Sequencing of two CDSs in African green monkey, three in gorilla, and three in chimpanzee allowed us to look for *ASPM* polymorphism in those species (see Figure 3E). Human polymorphism data from *ASPM* mutant haplotypes are not representative of wild-type variation so were not used in these comparisons. For African green monkey, five synonymous and five nonsynonymous changes were found between two sequences. The gorilla and chimpanzee CDSs in particular showed an apparently high degree of replacement polymorphism. Gorilla polymorphism included 35 point mutations (15 silent mutations and 21 replacements). Chimpanzee sequences differed in five synonymous and 11 nonsynonymous sites. In order to interpret this seemingly high level of observed polymorphism, intraspecific diversity was compared to interspecific diversity using the McDonald and Kreitman test (McDonald and Kreitman 1991). In the case of chimpanzee polymorphism compared to divergence with human, we could not reject the null hypothesis that polymorphism and divergence between species were significantly different (William's adjusted G statistic = 0.083, chi-square with 1 d.f., not significant; values based on PAML-generated Ka and Ks values using the free ratio model). Gorilla polymorphism was compared to divergence between the gorilla common ancestor and the human–chimpanzee common ancestor. In this case we can reject the null hypothesis (William's adjusted G statistic = 122.45, chi-square with 1 d.f., *p* < 0.001) to conclude that the pattern of gorilla polymorphism is therefore different from the divergence pattern. Indeed gorilla polymorphism is less than variation resulting from divergence: within species, the ω ratio is 1.43 for gorillas compared to 2.2 for the divergence between the gorilla common ancestor and the human–chimpanzee common ancestor. Intraspecific variation, although seemingly unusual in showing so many replacement substitutions in both chimpanzee and gorilla, is less than or in line with what we have observed for *ASPM* divergence between species. Therefore, relaxation of selection cannot explain the high nonsynonymous/synonymous substitution ratios among African hominoids, further supporting the idea that adaptation has occurred in *ASPM.*

## Discussion

In this study, we applied TAR cloning technology to investigate molecular evolution of the *ASPM* gene, which is involved in determining the size of the human brain and in which mutations lead to MCPH. The *ASPM* homologue in the fruit fly is essential for spindle function, suggesting a role for this gene in normal mitotic divisions of embryonic neuroblasts. Complete gene homologues from five primate species were isolated and sequenced. In agreement with the predicted critical role of *ASPM* in brain development, both coding and noncoding regions of *ASPM* homologues showed a marked degree of conservation in humans, other hominoids, and Old World monkeys. The differences found in noncoding regions were small insertions/deletions and lineage-specific insertions of evolutionarily young *Alu* elements into introns.

Analysis of nonsynonymous/synonymous substitution ratios indicates different rates of evolution along the ASPM protein: part of ASPM evolved under positive selection while other parts were under negative (purifying) selection in human and African ape lineages. Such “mosaic” selection has been previously described for other proteins (Endo et al. 1996; Crandall et al. 1999; Hughes 1999; Kreitman and Comeron 1999). When our work was completed, the paper by Zhang supporting accelerated evolution of the human ASPM was issued (Zhang 2003). However, because the author did not analyze the gorilla gene homologue, he concluded that accelerated sequence evolution is specific to the hominid lineage. Our finding that selection on *ASPM* begins well before brain expansion suggests that the molecular evolution of *ASPM* in hominoids may indeed be an example of a molecular “exaptation” (Gould and Vrba 1982), in that the originally selected function of *ASPM* was for something other than large brain size. 

In the case of *ASPM,* rapidly evolving residues are mainly concentrated in the IQ repeat domain containing multiple IQ motifs, which are calmodulin-binding consensus sequences. While there is no direct evidence yet, it is likely that the function of human *ASPM* is modulated through calmodulin or calmodulinlike protein(s). Previous interspecies comparisons of ASPM proteins have shown a consistent correlation of greater protein size with larger brain size mainly because of the number of IQ repeats (Bond et al. 2002). For example, the *asp* homologue of the nematode Caenorhabditis elegans contains two IQ repeats, the fruit fly*—*24 IQ repeats, and the mouse—61 IQ repeats, and there are 74 IQ repeats in humans (Bond et al. 2002). *ASPM* homologues in the nonhuman primates examined here contain the same number of IQ repeats as human, supporting the idea that repeat expansion occurred prior to the anthropoid divergence (which gave rise to New World monkeys, Old World monkeys, and hominoids) and possibly even earlier in primate evolution. IQ motifs are seen in a wide variety of proteins, but the ASPM proteins in primates are unique, because they have the largest known number of IQ repeats. Given the proposed role of *ASPM* in regulating divisions of neuronal progenitors, both the number of repeats and the particular amino acid substitutions in the IQ repeats may be strongly related to brain evolution.

Human *ASPM* gene mutations which lead to MCPH provide a direct link between genotype and phenotype. *ASPM* is yet another example on the growing list of positively selected genes that show both accelerated evolution along the human lineage and involvement in simple Mendelian disorders (Clark et al 2003). However, *ASPM* is unique because its distinctive pattern of accelerated protein evolution begins several million years prior to brain expansion in the hominid lineage. Absolute brain size in orangutans (430 g in males; 370 g in females) is barely different from that in gorillas (530 g in males; 460 g in females) and common chimpanzees (400 g in males; 370 g in females) (Tobias 1971), yet accelerated *ASPM* evolution began in the common ancestor of gorillas, chimpanzees, and humans, approximately 7–8 million years ago. Only much later did brain expansion begin in hominids, starting at 400–450 g roughly 2–2.5 million years ago and growing to its final current size of 1350–1450 g approximately 200,000–400,000 years ago (Wood and Collard 1999). Therefore genotypic changes in *ASPM* preceded marked phenotypic changes in hominoid brain evolution, at least at the level at which they have currently been studied. The molecular changes in *ASPM* may predict the existence of differences in early neurogenesis between orangutans, on the one hand, and gorillas, chimpanzees, and humans, on the other, which may manifest as more subtle differences in brain anatomy than gross changes in brain volume.

How might evolutionary changes in the ASPM protein affect cerebral cortical size? One potential mechanism might be that changes in ASPM induce changes in the orientation of the mitotic spindle of neuroblasts. Normally, neural precursor cells can have mitotic spindles oriented parallel to the ventricle or perpendicular to the ventricle. Mitoses in which daughter cells are oriented next to one another at the ventricular zone are typically “symmetric” in that a single progenitor cell generates two progenitor cells, causing exponential expansion of the progenitor pool. In contrast, mitoses that generate daughter cells that are vertically arranged are typically “asymmetric” so that one daughter cell becomes a postmitotic neuron, whereas the other daughter cell remains as a progenitor, causing only a linear increase in cell number. Control of this proliferative symmetry can cause dramatic alterations in cerebral cortical size (Chenn and Walsh 2002), and so changes in ASPM could regulate cortical size by making subtle changes in spindle orientation. Alternatively, evolutionary changes in ASPM may not themselves have led to increase in the size of the brain, but instead perhaps ASPM might be essential to insure faithful DNA replication and proper chromosome segregation. In rodents, a surprising number of cerebral cortical neurons are aneuploid (Rehen et al. 2001). Perhaps directed selection of specific domains of ASPM helps insure faithful chromosome segregation to allow a larger number of cerebral cortical neurons to be formed without an unduly high incidence of chromosome aneuploidy.

Functional genomics studies are clearly needed to elucidate the exact nature of the molecular mechanisms affected by *ASPM* gene evolution in hominoids. Here, we have demonstrated the utility of TAR cloning for evolutionary sequence comparisons among humans and other primates. In addition, the *ASPM* TAR clones isolated in these studies could provide valuable reagents for studying *ASPM* gene regulation in its natural sequence context. Overall, we anticipate this technology will be extremely useful in studying the evolution of other genes that may be responsible for uniquely human traits.

### Note

The related paper by Evans et al. (2004) was published in *Human Molecular Genetics* shortly after this paper was submitted.

## Materials and Methods

### 

#### TAR cloning of the *ASPM* gene homologues by in vivo recombination in yeast

To isolate the full-size *ASPM* gene from the human *(Homo sapiens),* chimpanzee *(Pan troglodyte*s*),* gorilla *(Gorilla gorilla),* orangutan *(Pongo pygmaeus),* and rhesus macaque *(Macaca mulatta)* genomes, a TAR vector containing two unique hooks was constructed. Two targeting sequences were designed, 131 bp 5′ and 151 bp 3′, from the available human genomic sequence of *ASPM* (positions 155,758–155,888 and 92,922–93,071 in the BAC RP11–32D17 [GI:16972838]). The targeting sequences were PCR amplified from the genomic DNA using two specific primers (Table S2). PCR products were cloned into a polylinker of the basic TAR vector pVC604 as *Apa*I–*Sal*I and *Sal*I–*Xba*I fragments. Before transformation experiments, the TAR cloning vector was linearized with *Sal*I to release targeting hooks. Genomic DNA samples were prepared from chimpanzee, gorilla, orangutan, and rhesus macaque fibroblast culture cell lines (Coriell Institute for Medical Research, Camden, New Jersey, United States) in agarose plugs. Spheroplast transformation experiments were carried out as previously described in Kouprina and Larionov (1999). To identify clones positive for *ASPM*, yeast transformants were examined by PCR using diagnostic primers specific for exon 2 and exon 27 of *ASPM* (Table S2). Integrity of yeast artificial chromosomes (YACs) and the issue of their stability during propagation in yeast were examined. DNA was isolated from ten subclones carrying the *ASPM* YACs for each primate, and their size was analyzed by *Not*I digestion followed by CHEF. Each subclone carried a YAC of similar size, indicating that these clones were stable in yeast. *Alu* profiles of the clones were checked by *Taq*I digestion of 1 μg of total yeast DNA isolated from transformants. Samples were run by electrophoresis, transferred to a nylon membrane, and hybridized with an *Alu* probe. YACs were retrofitted into bacterial artificial chromosomes (BACs) by homologous recombination in yeast using a BAC/Neo^R^ retrofitting vector, BRV1, and then transformed into a *recA* DH10B E. coli strain (Kouprina and Larionov 1999). Before sequencing, the integrity of inserts in BACs was confirmed by *Not*I, *Hin*dIII, *Eco*RI, and *Pst*I digestions. The promoter regions of the chimpanzee, gorilla, orangutan, and rhesus macaque (approximately 3.2 kb) and exon 18 of the red-chested mustached tamarin *(Saguinus labiatus)* and black-handed spider monkey *(Ateles geoffroyi)* (approximately 4.7 kb) were PCR amplified using a pair of specific primers (Table S2) from primate genomic DNAs (Coriell Institute for Medical Research) and then TA-subcloned for further sequencing.

#### RT-PCR of *ASPM* coding regions

RNAs were extracted from primate cell lines (African green monkey *[Cercopithecus aethiops]* kidney, COS-7 [American Type Culture Collection, Manassas, Virginia, United States], chimpanzee peripheral lymphoblast, EB176 [JC], and gorilla peripheral lymphoblast, EB [JC] [European Collection of Cell Cultures, Wiltshire, United Kingdom]) using TRIzol reagent (Invitrogen, Carlsbad, California, United States). Reverse transcription and 5′- and 3′-RACE reactions were performed using SMART RACE cDNA Amplification Kit (BD Biosciences, San Jose, California, United States). 

#### Sequencing

Chimpanzee, gorilla, orangutan, and rhesus macaque TAR clones containing full-size *ASPM* genes were directly sequenced from BAC DNAs (Polushin et al. 2001). Forward and reverse sequencing of the promoter and exon 18 as well as fragments of coding regions of the *ASPM* homologues were run on a PE-Applied Biosystem 3100 Automated Capillary DNA Sequencer (Applied Biosystems, Foster City, United States). Primer pairs for cDNA sequencing were designed based on the human *ASPM* mRNA sequence. Primer sequences are available upon request. All sequences were named and numbered according to the clone/accession identifier.

#### Sequence analysis

Genomic sequences were aligned using MAVID (http://baboon.math.berkeley.edu/mavid/) (Bray and Pachter 2004); proteins and protein-coding DNA sequences were aligned by DIALIGN2.1 (http://bibiserv.techfak.uni-bielefeld.de/dialign/) (Morgenstern 1999). Alignments were manually edited in the SEAVIEW editor (http://pbil.univ-lyon1.fr/software/seaview.html) (Galtier et al. 1996). We have used a number of programs from the EMBOSS package (http://www.hgmp.mrc.ac.uk/Software/EMBOSS/) for sequence analysis. Short nucleotide patterns associated with genome rearrangements were searched using FUZZNUC (EMBOSS). We searched for the following recombinogenic motifs: chi-like octamer (GCWGGWGG), immunoglobulin heptamer (GATAGTG), translin (ATGCAGN(0,4)GCCCWSW and GCNCWSCTN(0,2)GCCCWSSW), topoisomerase II (RNYNNCNNGYNGKNYNY), topoisomerase IId (GTNWAYATTNATNNR), topoisomerase IIi (YYCNTASYGGKYYTNNC), and V(D)J recombinase (CACAGTGN(12/23)ACAAAAACC). For short or highly ambiguous patterns (topo-isomerase II), no mismatches were allowed; for longer motifs (translin, V(D)J recombinase) up to two mismatches were permitted. Prediction of CpG islands was performed by CPGPLOT (EMBOSS) with default parameters (length ≥ 200; CpG/GpC ≥ 0.6; GC ≥ 0.5). CENSOR (http://www.girinst.org/Censor_Server-Data_Entry_Forms.html) (Jurka et al. 1996) and REPEATMASKER (http://repeatmasker.genome.washington.edu/cgi-bin/RepeatMasker; developed by A.F.A. Smit and P. Green) were used for identification of repetitive elements. Minisatellites were detected by TANDEM REPEAT FINDER (Benson 1999). *ASPM* segmental duplications in the human genome were detected by local BLAT searches (http://genome.ucsc.edu/cgi-bin/hgBlat) (Kent 2002). First, we used *ASPM* genomic sequences with all repeats masked to detect segmental duplications. Full-size duplications were then obtained by BLAT alignment with full (i.e., repeat-containing) *ASPM* sequence. Primate CDSs were deduced from the *ASPM* gene alignment with human sequences. Synonymous and nonsynonymous substitutions were detected by SNAP (http://www.hiv.lanl.gov/content/hiv-db/SNAP/WEBSNAP/SNAP.html). Codon maximum likelihood (ML) in CODEML in PAML v. 3.13 (http://abacus.gene.ucl.ac.uk/software/paml.html) (Yang 1997) has been applied for reconstruction of phylogenetic trees, reconstruction of ancestral sequences, and detection of positive selection. Branch lengths and ancestral sequences were reconstructed using a free ω ratio for individual branches. The methodology of likelihood ratio tests is described elsewhere (Yang 1998). For large alignments, the initial input trees for PAML were estimated by ML implemented in PHYLO_WIN (http://pbil.univ-lyon1.fr/software/phylowin.html) (Galtier et al. 1996). Segmental duplications were clustered by a neighbor-joining method implemented in the same program. Distance measurements for examining intraspecific/interspecific diversity were calculated in PAUP (Swofford, D. L. 2003. PAUP v. 4.0b10; Sinauer Associates, Sunderland, Massachusetts, United States; http://paup.csit.fsu.edu/index.html) and corrected for multiple substitutions using the Tamura-Nei algorithm.

## Supporting Information

**Commentary**

Selection operating on codon usage may increase the ω ratio by lowering the rate of synonymous substitutions (Sharp and Li 1987, 1989). Therefore, we tested the correlations between the CAI (Sharp and Li 1987) and the rate of synonymous substitutions (Ks). We found no significant association between the tested variables. Moreover, interspecies comparisons disclosed that CAI is nearly identical for all compared species, and no CAI increase over other species was detected for human or gorilla (data not shown). On the other hand, there was a significant negative correlation between CAI and both protein and DNA identity. A partial correlation analysis revealed that the significant positive linear correlation between Ka and CAI was merely caused by the strong negative correlation of Ka with DNA and protein identity. When we controlled for identity, the correlation between Ka and CAI disappeared (data not shown). These results may indicate that at positively selected sites, protein changes are preferred over optimization of codon usage, and thus mutations causing amino acid replacements do not immediately produce optimal codons. It should be noted that selection on codon usage seems to be generally relaxed in mammals (Duret and Mouchiroud 2000). Mammalian codon usage as well as the rate of nonsynonymous substitutions can be potentially biased by selection favoring a high GC content (or even saturation by G and C) at the third codon positions (GC3) (Bernardi and Bernardi 1985; Aota and Ikemura 1986). However, *ASPM* is an AT-rich gene (GC content 36.4%–37%) and, as expected (Bernardi and Bernardi 1985; Aota and Ikemura 1986), the third codon positions are also AT-rich (GC3 content, 30.6%–31.4%) and thus far from saturation. In summary, neither the codon bias nor selection on the third codon seems to strongly influence the synonymous rate Ks. Therefore the high Ka/Ks ratio in human and gorilla is likely to be the product of adaptive evolution.

Figure S1 Recombination Breakpoints in the Orangutan-Specific 818-bp-Long Deletion Both orangutan breakpoints are located within 5′ portions of two *Alu* elements. The sequence conservation is marked by different shades of gray. Both *Alu* elements are compared to their corresponding *AluSp* and *AluSz* subfamily consensus sequences. Gorilla, chimpanzee, and human sequences located 1 bp downstream of the 5′ breakpoint share a perfect match with the chi-like octamer consensus sequence GCWGGWGG (first box, positions matching the chi consensus are shown in black). On the other hand, the 3′ breakpoint sequences are diverged from the chi consensus (second box). Both *Alu* elements in the alignment are shown from the first position and end at the same position, and thus positions in one element correspond to positions in the other *Alu* copy. As can be seen, the breakpoint position in the first *AluSp* repeat exactly corresponds to the breakpoint position within the second *AluSz* element, suggesting homologous recombination between the two repeats.
(163 KB PDF).

Figure S2 Segmental Duplications of the Fourth Internal Intron From left to right: phylogeny, chromosomal position, band name, identity to *ASPM* segment (percent same), and a schematic alignment of segmental duplications. The *ASPM* segment (black) shares similarity with 24 segmental duplications that contain additional sequences and are present on several human chromosomes. The *ASPM* copy and three duplications on Chromosome 7 share the same *L1P4* terminal insertion, which is absent from all other duplications. The tree on the left shows evolutionary relationships among the duplications estimated by the neighbor-joining method.
(169 KB PDF).

Figure S3 Comparison of Mouse and Human ASPM Proteins The amino acid identity in the conserved regions is 85.44%, 49.39%, and 68.74% for exon 3, exon 4, and the IQ domain, respectively. In addition, while the alignment of conserved regions is completely gap-free, the variable domains exhibit several gaps including a large deletion in the mouse IQ domain (human positions 2655–2943).
(97 KB PDF).

Table S1 Primers Used in This Work Upper case letters indicate sequences homologous to *ASPM* and lower case letters indicate cloning sites.
(118 KB PDF).

Table S2 CDS and Protein Correlations All correlations were obtained for the same 100-amino-acid-/300-nucleotide-long, nonoverlapping windows. The first value shows the correlation coefficient; *p*-value is in parentheses. The section over the diagonal is calculated using the Pearson (linear) correlation coefficient; under the diagonal are correlations obtained using the Spearman's rank coefficient—nonparametric). Nontrivial or interesting significant correlations are shown in bold and italics. The CAI represents the mean for all species (the CAI values are nearly identical for individual species). The ω ratio, Ka, and Ks (rows/columns 2, 3, and 4) correspond to all branches of the phylogenetic tree. They were obtained using a ML model with one fixed ω ratio for all branches.

### **Accession Numbers**

The GenBank (http://www.ncbi.nlm.nih.gov/Genbank/) accession number for the human *ASPM* mRNA sequence used in this study is NM_018136. The sequence data from chimpanzee, gorilla, orangutan, and rhesus macaque full-length *ASPM* have been submitted to GenBank under accession numbers AY497016, AY497015, AY497014, and AY497013. The sequence data from chimpanzee, gorilla, and African green monkey *ASPM* cDNA have been submitted to GenBank under accession numbers AY508452, AY508451, and AY486114. The sequence data from spider monkey and tamarin exon 18 have been submitted to GenBank under accession numbers AY497017 and AY497018.

## Abbreviations

BAC: bacterial artificial chromosome
CAI: codon adaptation index
CDS: coding sequence
MCPH: primary microcephaly
ML: maximum likelihood
SNP: single nucleotide polymorphism
TAR: transformation-associated recombination
YAC: yeast artificial chromosome
