# Supplementary material for: Accelerated Evolution of the ASPM Gene Controlling Brain Size Begins Prior to Human Brain Expansion
Source: PLoS Biol. 2004 Mar 23;2(5):e126. doi: 10.1371/journal.pbio.0020126 (PMC374243; doi:10.1371/journal.pbio.0020126)
Supplement: Figure S1 — Both orangutan breakpoints are located within 5′ portions of two Alu elements. The sequence conservation is marked by different shades of gray. Both Alu elements are compared to their corresponding AluSp and AluSz subfamily consensus sequences. Gorilla, chimpanzee, and human sequences located 1 bp downstream of the 5′ breakpoint share a perfect match with the chi-like octamer consensus sequence GCWGGWGG (first box, positions matching the chi consensus are shown in black). On the other hand, the 3′ breakpoint sequences are diverged from the chi consensus (second box). Both Alu elements in the alignment are shown from the first position and end at the same position, and thus positions in one element correspond to positions in the other Alu copy. As can be seen, the breakpoint position in the first AluSp repeat exactly corresponds to the breakpoint position within the second AluSz element, suggesting homologous recombination between the two repeats. (163 KB PDF). [file pbio.0020126.sg001.pdf]

SUPPLEMENTARY MATERIAL

Figure 1S Recombination breakpoints in the orangutan-specific 818bp-long deletion

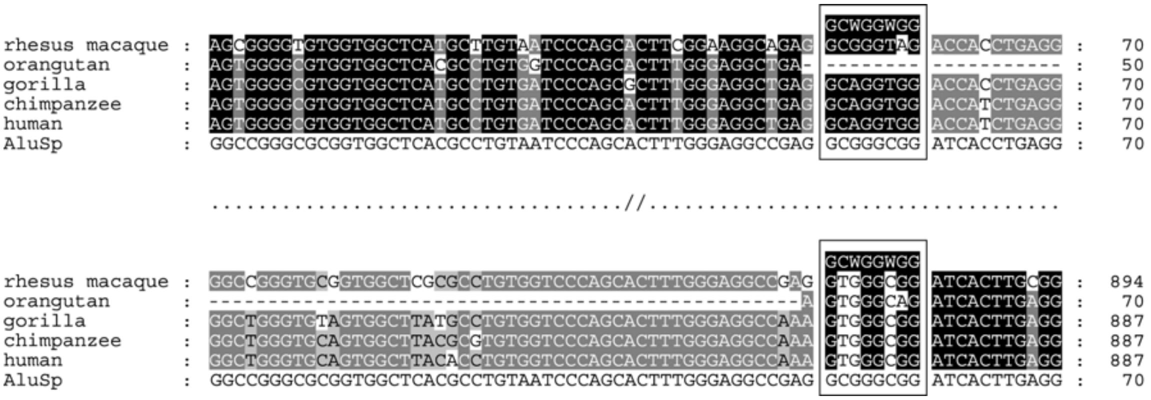

Both orangutan breakpoints are located within 5' portions of two *Alu* elements. The sequence conservation is marked by different shades of gray. Both *Alu* elements are compared to their corresponding *AluSp* and *AluSz* subfamily consensus sequences. Gorilla, chimpanzee, and human sequences located 1bp downstream of the 5' breakpoint share a perfect match with the chi-like octamer consensus sequence GCWGGWGG (first box, positions matching the chi consensus are shown in black). On the other hand, the 3' breakpoint sequences are diverged from the chi consensus (second box). Both *Alu* elements in the alignment are shown from the first and end at the same position and thus positions in one element correspond to position in the other *Alu* copy. As can be seen, the breakpoint position in the first *AluSp* repeat exactly corresponds to the breakpoint position within the second *AluSz* element, suggesting homologous recombination between the two repeats.
