# Supplementary material for: Accelerated Evolution of the ASPM Gene Controlling Brain Size Begins Prior to Human Brain Expansion
Source: PLoS Biol. 2004 Mar 23;2(5):e126. doi: 10.1371/journal.pbio.0020126 (PMC374243; doi:10.1371/journal.pbio.0020126)
Supplement: Figure S2 — From left to right: phylogeny, chromosomal position, band name, identity to ASPM segment (percent same), and a schematic alignment of segmental duplications. The ASPM segment (black) shares similarity with 24 segmental duplications that contain additional sequences and are present on several human chromosomes. The ASPM copy and three duplications on Chromosome 7 share the same L1P4 terminal insertion, which is absent from all other duplications. The tree on the left shows evolutionary relationships among the duplications estimated by the neighbor-joining method. (169 KB PDF). [file pbio.0020126.sg002.pdf]

Figure 2S Segmental duplications of the fourth internal intron

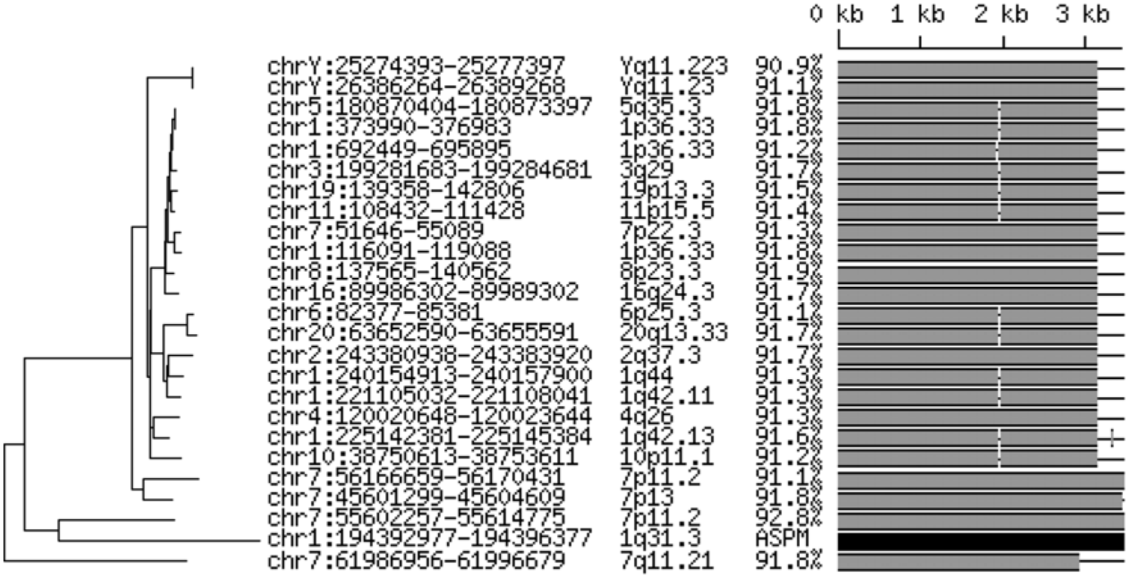

From left to right: phylogeny, chromosomal positions, cytogenetic band name, identity to *ASPM* segment (%), and a schematic alignment of segmental duplications. The *ASPM* segment (black) shares similarity with 24 segmental duplications that contain additional sequences and are present on several human chromosomes. The *ASPM* copy and three duplications on chromosome 7 share the same L1P4 terminal insertion, which is absent from all other duplications. The tree on the left shows evolutionary relationships among the duplications estimated by the neighbor-joining method.
