# Supplementary material for: Accelerated Evolution of the ASPM Gene Controlling Brain Size Begins Prior to Human Brain Expansion
Source: PLoS Biol. 2004 Mar 23;2(5):e126. doi: 10.1371/journal.pbio.0020126 (PMC374243; doi:10.1371/journal.pbio.0020126)
Supplement: Figure S3 — The amino acid identity in the conserved regions is 85.44%, 49.39%, and 68.74% for exon 3, exon 4, and the IQ domain, respectively. In addition, while the alignment of conserved regions is completely gap-free, the variable domains exhibit several gaps including a large deletion in the mouse IQ domain (human positions 2655–2943). (97 KB PDF). [file pbio.0020126.sg003.pdf]

Figure 3S Comparison of mouse and human ASPM proteins

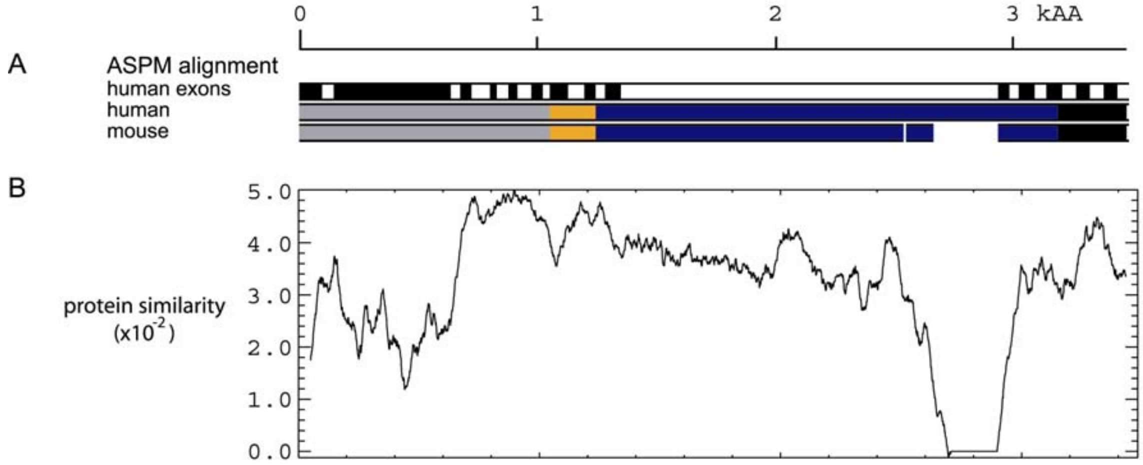

A. Schematic representation of the human-mouse protein alignment. Positions of exons in the human coding sequence are drawn in the first block. To separate individual exons, odd exons are colored in black and the even ones in white. The second and third plots show the protein alignment, white segments mark gaps. The putative microtubule-binding domain is in gray, calponin-homology domain - in orange, IQ repeats - in blue, and terminal domain - in black. **B.** Similarity plot for the human-mouse protein alignment. Plotcon (EMBOSS) was used with a default BLOSUM62 substitution matrix and a 100aa-long window.

Commentary:

The amino acid identity in the conserved regions is 85.44%, and 49.39% and 68.74% for exons 3-4 and the IQ domain, respectively. In addition, while the alignment of conserved regions is completely gap-free, the variable domains exhibit several gaps including a large deletion in the mouse IQ domain (human positions 2655-2943).
