# Supplementary material for: Accelerated Evolution of the ASPM Gene Controlling Brain Size Begins Prior to Human Brain Expansion
Source: PLoS Biol. 2004 Mar 23;2(5):e126. doi: 10.1371/journal.pbio.0020126 (PMC374243; doi:10.1371/journal.pbio.0020126)
Supplement: Table S1 — Upper case letters indicate sequences homologous to ASPM and lower case letters indicate cloning sites. (118 KB PDF). [file pbio.0020126.st001.pdf]

Table 1S Primers used in this work

Primer	Sequence
**Hook**	
Ex1F/Ex1R	5'-agctatgcgtcgacTTCGCCATGGCAGATTCGAG-3'/5'-atgctctagaTCACTCCCACGACCTCTACA-3'
3F/3R	5'-atgcgggcccTGGGCAGACTTTGAGACTCT-3'/5'-agctatgcgtcgacAGTTATACACCACAGACCTGA-3'
**Diagnostic**	
DGex2-1F/DGex2-1R	5’-GGCCAAAGAGGAGAGAATAG-3’/5’-CTGACTCTGATTCTAGATCC-3’
DGex2-2F/DGex2-2R	5’-CCTAAGAGACGTCCAATAC-3’/5’-TGTCGAAGAGGGTGTTACCT-3’
DGex27-F/DGex27-R	5’-GGAGCTTTTGCAGATATACCG-3’/5’-GTCATAGACTTAAGACCACAG-3’
**Promoter**	
Pro-F/Pro-R1	5'-TTCGCCATGGCAGATTCGAG-3'/5'-ATGTCTGGTGGTCCACAGTG-3'
**Exon 18**	
Ex18-F1/Ex18-R2	5’-GGATATTGGAGAAGATATTC-3’/5’-CCTGAAACTTCCGTTTCTGT-3’

Upper case letters indicate sequences homologous to *ASPM* and lower case letters indicate cloning sites.
