# Supplementary material for: Accelerated Evolution of the ASPM Gene Controlling Brain Size Begins Prior to Human Brain Expansion
Source: PLoS Biol. 2004 Mar 23;2(5):e126. doi: 10.1371/journal.pbio.0020126 (PMC374243; doi:10.1371/journal.pbio.0020126)
Supplement: Table S2 — All correlations were obtained for the same 100-amino-acid-/300-nucleotide-long, nonoverlapping windows. The first value shows the correlation coefficient; p-value is in parentheses. The section over the diagonal is calculated using the Pearson (linear) correlation coefficient; under the diagonal are correlations obtained using the Spearman's rank coefficient—nonparametric). Nontrivial or interesting significant correlations are shown in bold and italics. The CAI represents the mean for all species (the CAI values are nearly identical for individual species). The ω ratio, Ka, and Ks (rows/columns 2, 3, and 4) correspond to all branches of the phylogenetic tree. They were obtained using a ML model with one fixed ω ratio for all branches. [file pbio.0020126.st002.pdf]

Table 2S CDS and proteins correlations

	Mean CAI	ω ratio	Ka	Ka	DNA identity	Protein identity
**Mean CAI**		0. 251 (p=0.152)	***0. 339 (p=0.05)***	-0. 038 (p=0. 832)	***-0. 517 (p=0.002)***	***-0. 352 (p=0. 041)***
**ω ratio**	0. 349 (p=0. 043)		0. 654 (p=0.000)	-0.638 (p=0.000)	-0. 246 (p=0. 162)	***-0. 556 (p=0. 001)***
**Ka**	0. 325 (p=0. 061)	0. 707 (p=0.000)		-0. 020 (p=0. 909)	-0. 583 (p=0. 000)	-0. 777 (p=0. 000)
**Ka**	-0. 107 (p=0. 547)	-0. 655 (p=0.000)	0. 011 (p=0. 950)		-0. 270 (p=0. 122)	-0. 006 (p=0. 972)
**DNA identity**	***-0. 405 (p=0.017)***	***-0. 408 (p=0. 016)***	-0. 652 (p=0.000)	-0. 140 (p=0. 428)		0. 868 (p=0.000)
**Protein identity**	***-0. 312 (p=0. 072)***	***-0. 575 (p=0.000)***	-0. 756 (p=0.000)	-0. 018 (p=0. 919)	0. 924 (p=0.000)	

All correlations were obtained for the same 100aa/300nt long, nonoverlapping windows. The first value shows the correlation coefficient; p-value is in parenthesis. The section over the diagonal is calculated using the Pearson (linear) correlation coefficient; under the diagonal are correlations obtained using the Spearman's rank coefficient - nonparametric). Nontrivial or interesting significant correlations are shown in bold and italics. The codon adaptation index (CAI) represents mean for all species (the CAI values are nearly identical for individual species). The ω ratio, Ka and Ks (rows/columns 2, 3 and 4) correspond to all branches of the phylogenetic tree. They were obtained using a ML model with one fixed ω ratio for all branches.

Commentary:

Selection operating on codon usage may increase the ω ratio by lowering the rate of synonymous substitutions (Sharp and Li, 1987b; 1989). Therefore we tested the correlations between the codon adaptation index (CAI, Sharp and Li, 1987a) and the rate of synonymous substitutions (Ks). We have found no significant association between the tested variables. Moreover, interspecies comparisons disclosed that CAI is nearly identical for all compared species and no CAI increase over other species was detected

for human or gorilla (not shown). On the other hand, there is a significant negative correlation between CAI and both protein and DNA identity. A partial correlation analysis revealed that the significant positive linear correlation between Ka and CAI is merely caused by the strong negative correlation of Ka with DNA and protein identity. When we controlled for identity, the correlation between Ka and CAI disappeared (not shown). These results may indicate that positively selected sites protein changes are more preferred than optimization of codon usage and thus mutations causing amino acids replacements do not immediately produce optimal codons. It should be noted that selection on codon usage seems to be generally relaxed in mammals (Duret & Mouchiroud, 2000). Mammalian codon usage as well as the rate of nonsynonymous substitutions can be potentially biased by selection favoring a high GC content (or even saturation by G and C) at the third codon positions (GC3; Bernardi and Bernardi, 1985; Aota and Ikemura, 1986). However, ASPM is an AT-rich gene (GC content 36.4-37%) and, as expected (Bernardi and Bernardi,1985; Aota and Ikemura, 1986), the third codon positions are also AT-rich (GC3 content 30.6-31.4%) and thus far from saturation. In summary neither the codon bias nor selection on the third codon seem to strongly influence the synonymous rate Ks. Therefore the high Ka/Ks ration in human and gorilla are likely to be product of adaptive evolution.

Aota, S. and Ikemura, T. (1986) Diversity in G + C content at the third position of codons in vertebrate genes and its cause. *Nucleic Acids Res*. **14**, 6345-6355.

Bernardi, G. and Bernardi, G. (1985) Codon usage and genome composition. *J. Mol. Evol.* **22**, 363-365.

Duret, L. & Mouchiroud, D. (2000) Determinants of substitution rates in mammalian genes: expression pattern affects selection intensity but not mutation rate. *Mol. Biol. Evol.* **17**, 68-74.

Sharp, P.M. and Li, W.H. (1987) The rate of synonymous substitution in enterobacterial genes is inversely related to codon usage bias. *Mol. Biol. Evol*. **4**, 222-230.

Sharp, P.M. and Li, W.H. (1989) On the rate of DNA sequence evolution in Drosophila. *J. Mol. Evol.* **28**, 398-402.
